# Strategies for Teaching Linguistic Preparedness for Physicians: Medical Spanish and Global Linguistic Competence in Undergraduate Medical Education

**DOI:** 10.1089/heq.2019.0029

**Published:** 2019-07-01

**Authors:** Pilar Ortega, Norma Pérez, Brenda Robles, Yumirle Turmelle, David Acosta

**Affiliations:** ^1^ Department of Emergency Medicine, College of Medicine, University of Illinois, Chicago, Illinois.; ^2^ Department of Medical Education, College of Medicine, University of Illinois, Chicago, Illinois.; ^3^ Special Programs, School of Medicine, Hispanic Center of Excellence, University of Texas Medical Branch, Galveston, Texas.; ^4^ Language Interpreters Program, National Institutes of Health Clinical Center, Bethesda, Maryland.; ^5^ Washington University St. Louis School of Medicine, St. Louis, Missouri.; ^6^ Association of American Medical Colleges, Washington, District of Columbia.

**Keywords:** language concordance, medical Spanish, patient-physician communication, Hispanic/Latino health, linguistic proficiency, medical interpreters, clinical communication skills

## Abstract

In accordance with Liaison Committee on Medical Education (LCME) curriculum content standards, medical schools are expected to teach physician communication skills and cultural competence. Given the sustained U.S. Spanish-speaking population growth, importance of language in diagnosis, and benefits of patient–physician language concordance, addressing LCME standards equitably should involve linguistic preparedness education. The authors present strategies for implementation of linguistic preparedness education in medical schools by discussing (1) examples of institutional approaches to dedicated medical Spanish courses that meet best practice guidelines and (2) a partnership model with medical interpreters to implement integrated global linguistic competencies in undergraduate medical curricula.

## Introduction

The Liaison Committee on Medical Education (LCME) sets curriculum content standards for undergraduate medical education to ensure standardization of educational content for United States (U.S.) medical school graduates. In accordance with standards pertaining to communication skills and cultural competence of graduating medical students,^1^ medical schools are expected to provide education on communication skills pertinent to providing quality care for the U.S. patient population. Given the heterogeneous and dynamic linguistic profile of U.S. patients, communication skills education in medical schools should address these demographic realities to ensure health equity for all. Further, the Association of American Medical Colleges (AAMC) includes within its diversity and inclusion mission the objective to grow a diverse and culturally prepared health workforce by improving integration of public health concepts into medical education, supporting inclusion of social factors in health within medical education programs.^2^

Language alone, through medical history taking, has long been demonstrated to be sufficient in making a diagnosis in a majority (75%) of cases,^3^ and linguistic differences have been shown to lead to exacerbation of health disparities for the underserved Hispanic/Latino population.^4–7^ Prior national regulations and efforts have primarily focused on interpreter services only^8,9^ rather than on simultaneously enhancing language-concordant, direct patient–physician communication through medical education and assessment.^10^ Moreover, U.S. population trends demonstrate continued growth of Spanish use nationwide,^11^ yet patient–physician communication in languages besides English and the non-English linguistic proficiency of medical students are not routinely included in medical education curricula or assessment processes.

We propose that meeting LCME curricular content standards for communication and cultural competence in medical education within a public health context should include two primary elements of linguistic preparedness for physicians: global linguistic competence education and dedicated medical Spanish courses, outlined in Table 1. We define global linguistic competence for physicians as the skills needed to communicate with patients of any linguistic preferences or needs. Dedicated medical Spanish courses in medical school are defined as courses that teach and assess learner ability to competently use Spanish in the practice of medicine for direct communication with patients and to self-assess limitations.

**Table 1.** Elements of Linguistic Preparedness Education for Physicians: Summary of Key Features and Recommended Curricular Placement

Elements of linguistic preparedness education for physicians	Key features	Curricular placement
Dedicated medical Spanish course	• Linguistic skills for communication with Spanish-speaking patients • Prerequisite: intermediate Spanish proficiency	• Variable placement according to institutional preference • Elective course for eligible students
Global linguistic competence education	• Generalizable skills for communication with all patients regardless of language • Prerequisite: none	• Throughout clinical skills and communication courses • Integrated for all students

Multiple examples exist of curricula for medical Spanish education, and a majority of medical schools report medical Spanish courses according to the most recent survey;^12^ although methods of implementation are variable, most reported courses lack assessment of student skills, posing patient safety risks,^13^ and a standardized curriculum tested at multiple centers has never been attempted. We will illustrate several examples of institutional approaches to dedicated medical Spanish courses that can form the basis of a standardized curricular model and propose a partnership model with professional medical interpreters to implement the concept of integrated global linguistic competencies in undergraduate medical education.

## Dedicated Medical Spanish Courses

In the absence of an existing standardized curricular structure or list of learner competencies or objectives, individual schools and/or instructors typically design their own medical Spanish programs. This presents a challenge for schools without existing programs since creating a course from scratch is a significant academic endeavor, and the lack of standardization results in a lack of uniformity at different institutions. We provide examples of curricular structure at three academic institutions that were designed according to best practice guiding principles established by Reuland et al.^14^ and represent variations in execution that can be replicated at other centers. A comparison of the programs is illustrated in Table 2.

**Table 2.** Comparison of Existing Medical Spanish Programs That Meet Reuland's Guiding Principles,^14^ But Differ in Execution

	University of Texas Medical Branch	University of Illinois at Chicago College of Medicine	Washington University School of Medicine
How is the longitudinal and multiple learning modalities principle achieved?	• Longitudinal principle: Online hybrid course available 24/7 and offered in the third or fourth year. • 40 h/week×4 weeks	• Longitudinal principle: Introductory medical Spanish elements in the first/second year; Faculty-taught course during the third or fourth year. • 8 h/week×10 weeks, or 40 h/week×2 weeks	• Longitudinal principle: Language-focused course in the first year, semester 1; Culture-focused course in the first year, semester 2; Certification option in the second year; clerkship options in third and fourth years. • 1 h/week×20 weeks
	• Multiple modalities: Learning resource center, clinical modules, faculty-led videoconferences, language tools, and interactive videotaped encounter	• Multiple modalities: Patient interviews, SPs, faculty-taught lectures, self-study, case notes, online videos, and cultural projects	• Multiple modalities: Lecture, self-study, SPs, and clerkships at higher percentage Spanish-speaking sites
How is intermediate–advanced proficiency focus achieved?	• Minimum proficiency: Basic–intermediate oral proficiency • Assessment: Oral examination with course director using ACTFL standards	• Minimum proficiency: Intermediate oral proficiency • Assessment: Self-reported scale; faculty oral interview if level is uncertain	• Minimum proficiency: Advanced oral proficiency • Assessment: Oral interview with faculty
How is academic credit/status achieved?	• One elective credit (160 h of course time)	• Two elective credits (80 h of course time)	• One elective credit (10 h of course time) for language-focused course • One elective humanities credit (10 h of course time) for culture-focused course
How is curriculum integration achieved?	• Third and fourth years: Students self-schedule online modules over their selected 4 weeks.	• First and second years: Self-scheduled • Third and fourth years: 10-week course meets during evenings and can overlap with clinical clerkships; 2-week course is integrated into an elective block schedule.	• First and second years: Courses meet once per week to allow overlap with other classes • Third and fourth years: Student can select Spanish-speaking sites for clinical clerkships.
How is the communication skills focus achieved?	• Clinical modules focus on the health care communication skills by subject area. • Videotaped encounter focusing on communication skills is the primary endpoint.	• Class time is focused on role-play and verbal communication. • Student case notes are used to practice vocabulary at the patient literacy level rather than technical level. • SPs are trained to provide feedback on effective communication.	• Class time is focused on role-play and verbal communication. • Students are provided with real-time clinical opportunities to use Spanish, including volunteer clinic, teaching assistant for other students, SPs, and clinical clerkships.
How is the postcourse proficiency assessment achieved?	• 15 Inline components from the learning resource center and from the clinical modules, and final videotaped encounters.	• SP encounters with faculty scoring, learner self-scoring, SP scoring, and CIS scale.	• CCLA examination and SP encounters.

ACTFL, American Council on the Teaching of Foreign Languages; CCLA, Clinical Cultural and Linguistic Assessment; CIS, communication and interpersonal skills; SP, standardized patient.

## Curricular Models for Dedicated Medical Spanish

At the University of Texas Medical Branch (UTMB), a multidisciplinary hybrid course was designed to provide the student with basic structures of the Spanish language and the specialized medical vocabulary needed to communicate effectively with Spanish-speaking patients in a variety of clinical scenarios. Students actively self-manage their learning; the course is online, therefore modules can be completed during flexible times at the students' convenience. The course is divided into four main sections: (1) learning resource center, which provides a variety of learning experiences to help the student understand and apply course concepts, such as Spanish basics, grammar, anatomy, medical terminology, and culture. (2) Clinical modules, which contain the essentials of the Spanish language, culture, and the vocabulary needed to communicate effectively in a variety of health care situations, including greeting the patient, taking a full medical history, review of systems, physical examination, diagnosis, patient medication instructions, and special populations. (3) The third section provides the student easy access to basic language tools existing online. (4) The fourth component of the course is interactive, where students have to produce four taped encounters and, finally, a complete full medical history. Student assessment during the course includes self-reflection, peer and faculty feedback on videos, language assessment by faculty, and scoring on 15 inline components from the learning resource center and from the clinical modules.

The Clinical Conversational Spanish for Healthcare Professionals UTMB course has been in existence for 5 years, a total of 129 students have enrolled, and 100% have achieved successful course completion. Students report that course content is useful and relevant to clinics (72%), and 52% believe that clinical Spanish should be a mandatory course.^15^ Due to student popularity, the course is now accessible outside of UTMB through the Visiting Student Application Service (VSAS) of the AAMC.^16^

At the University of Illinois College of Medicine (UICOM), students hold peer-led faculty-supervised workshops on medical Spanish vocabulary and conversational skills during lunch as a preparatory step in first and second years of medical school. This initial exposure helps some students self-identify a need to improve their basic Spanish skills before qualifying for the third- and fourth-year Clinical Medical Spanish elective, for which an intermediate Spanish language requirement is implemented. The formal elective is designed to provide 2 weeks (80 h) of official credit spread out over a 10-week longitudinal elective, in which students have a 2-h evening lecture weekly, but have multiple self-study multimedia and grammar/vocabulary exercise components,^17^ patient interview requirements, mid- and end-course objective structured clinical examinations with trained standardized patients (SPs), and cultural research spread out over the course.^18^

The course is organized by organ–system (musculoskeletal, pulmonary, cardiovascular, gastrointestinal, endocrine, genitourinary, ophthalmologic and ear, nose, and throat, neurologic, psychiatric, and pediatric) with each week focusing on one subject and addressing class role-play, patient interviews, case write-ups, and development of culturally appropriate patient education materials. In the 10-week course, students are expected to overlap the medical Spanish elective with other clinical clerkships. In 4 years since course initiation, 158 students have enrolled and completed the Clinical Medical Spanish elective. Data analyzed for the first 2 years of implementation (51 respondents of 58 students, 88% response rate) showed that the self-rated comfort level with interviewing and examining Spanish-speaking patients significantly improved after the course, and the improvement in comfort level was sustained 1 year into residency training based on a follow-up survey completed by 64% of students. Eighty-nine percent of follow-up respondents reported that the elective was useful for their intern year, and 97.3% reported that they would recommend the course to other fourth-year medical students.^18^ To accommodate student need, a new 2-week intensive course will be piloted in the near future at UICOM, in which the curriculum will be condensed into a shorter time period, but students will dedicate to medical Spanish material full-time for the 2 weeks of the course.

At the Washington University School of Medicine in St. Louis, the medical Spanish curriculum is designed for advanced Spanish speakers, defined as native speakers or those with strong conversational skills. The goals of the program are for students to develop proficiency in conducting a clinical encounter in Spanish, emphasize the importance of both language and culture in patient–physician interactions, and obtain certification as a bilingual provider. The program is designed as a comprehensive and longitudinal experience to span all 4 years of medical school, with the classroom component primarily in year 1, certification in the summer after year 2, and clinical opportunities to serve Spanish-speaking patients in years 3 and 4. In the first semester of year 1, a 10-week selective is offered for clinical credit with weekly, 1-h, peer-led faculty-supervised classes to learn medical Spanish vocabulary and practice speaking during role-play. Two SP sessions in Spanish are included in this selective. During the second semester of year 1, a 10-week selective is offered as humanities credit with weekly 1-h classes to discuss cultural aspects of medical interaction, ethics, and professionalism. In year 2, students continue to practice their medical Spanish by required participation in a few of the following roles: teaching assistant for year 1 sessions, organizer for student group conversation sessions, on-call interpreter for a Spanish-speaking clinic, additional SP session participation, or grand rounds Spanish case presentations. At the end of year 2, students take the Clinical Cultural and Linguistic Assessment examination as part of their certification process. Years 3 and 4 have clerkships with incorporated bilingual components for eligible students.

## Global Linguistic Competence Education

The elements of global linguistic competence, which should be included as part of a longitudinal communication skills curriculum for all students regardless of Spanish proficiency or desire to enroll in a formal Spanish elective, should be as follows: understanding of linguistic diversity and the impact of language on population health; self-awareness of language proficiency and limitations in medical settings; competency in the use of a professional medical interpreter; and cultural issues in health that may confound successful medical communication. Critical communication elements related to language include cultural health beliefs, variations in health literacy, and patient distrust or other barriers to full communication with physicians, as well as the possibility of errors in communication due to limited physician Spanish skills or misunderstandings.

Medical school curricula pertaining to clinical communication skills may consider integration of global linguistic competence elements throughout existing programming without the need to add a separate course. If these components are only included in elective, dedicated medical Spanish courses, there would be a significant missed opportunity to expose students who do not qualify or desire to enroll in medical Spanish to these critical communication skills. For example, segments that can be considered throughout the curriculum include demonstration of correct and incorrect usage of professional interpreters, inclusion of readings or faculty lectures pertaining to linguistic minority patient populations relevant to a particular module (e.g., cardiovascular and pulmonary), discussion of cultural health beliefs or practices that may defy literal interpretation, and incorporation of SP cases that reflect underserved patients' social circumstances, including linguistic realities, and allow students to practice clinical skills and troubleshoot challenges.

It may be helpful to identify existing sources of expertise such as certified medical interpreters who could assist with specific components of linguistic competence education using an interdisciplinary approach. Similar partnerships could be sought with public health institutions, university foreign languages departments, or community organizations with educational missions such as *promotores de salud*.^19^

## Certified Medical Interpreters as a Partnership Model

Certified medical interpreters already serve as communication conduits in health care settings, assisting with verbal communication between providers and patients where language discordance is identified. Interpreters certified by accredited certification processes in the U.S.^20,21^ are skilled in medical vocabulary in source and target languages and adhere to a strict code of ethics founded on precision, boundaries, objectivity, confidentiality, and respect.^22^ In health care, the dynamic interdisciplinary partnership between interpreters and providers can also be extended to an educational partnership.

The certified medical interpreter's role is to maintain transparency, set boundaries, and recognize and intervene when a miscommunication may be taking place in a patient–provider encounter. Likewise, interpreters can help providers assess their needs and skills through observational progress assessments for medical Spanish learners as well as constructive feedback for medical students using interpreter services to improve the students' skills in effectively using language assistance tools, including onsite interpreters and remote interpreter devices. Although such technological advances are helpful in increasing access to professional interpreting, they require training to minimize perceived barriers to utilization and maximize effective communication.^23^

During dedicated medical Spanish courses, interpreters can potentially contribute during in-class role-play encounters, provide feedback on recorded or real-time simulated or SP interviews, or observe live clinical encounters in which the interpreter can serve as a safety net by intervening if miscommunication occurs. Medical interpreters can help students and providers determine their need for language assistance services in varying situations. For instance, providers with basic proficiency may be safely encouraged to engage in greetings and casual conversations as trust- and rapport-building opportunities; however, conversations about treatment plans, procedures, and informed consent require a much higher degree of medical Spanish skill and therefore a much lower threshold for utilization of a trained medical interpreter.^24^ In the case of intermediate or advanced proficiency, determining if the encounters will be general in scope or specialty-driven is also a fundamental consideration since the latter vocabulary may be out of the scope of even an advanced or native Spanish speaker. In addition, interpreters can participate in longitudinal, integrated global linguistic competence training in partnership with medical school faculty by acting as the interpreter during select SP encounters, providing sample clinical scenarios of culturally or linguistically complex cases, identifying materials that can be used for problem-based learning or role-play challenges (e.g., consent forms) in multilingual encounters, or giving guest lectures on cultural topics that may influence communication.

## Conclusions

Medical schools should consider strategies to incorporate the linguistic realities of U.S. patients as part of public health-conscious medical education. Addressing linguistic skills for medical students is supported by LCME core content standards and must adapt to demographic trends to ensure health equity for linguistic minorities and to address physician public health preparedness in an increasingly global U.S. population. Institutional endorsement of courses that address non-English language competencies is critical to the success and sustainability of educational programs and can be justified as part of existing medical education standards.

We propose that schools focus on two complementary approaches to linguistic needs of physicians in training: (1) dedicated medical Spanish courses for eligible students who desire to achieve and assess competency in direct patient–physician communication with Spanish-speaking patients and (2) integrated global linguistic competence education for all medical students within communication and clinical skills courses. Replication of an accepted curricular methodology that has been previously established should be considered to reduce the need for recreating new curricula for dedicated medical Spanish courses. Furthermore, interdisciplinary partnerships, such as collaboration with professional medical interpreters, can contribute to the cultural and self-awareness components critical for multilingual and monolingual physicians alike and should form part of a comprehensive longitudinal approach to linguistic competence in medical education.

Next steps should consider interinstitutional collaboration on curricular implementation and evaluation by means of data collection regarding student comfort, confidence, and skill demonstration. Varying aspects of implementation such as course duration, curricular placement, course faculty qualifications, and successful interdisciplinary partnership models should be studied to better understand best practices. In addition, continued engagement of institutional stakeholders such as administrators and leadership is important in assessing institutional priorities and considering integration of both dedicated medical Spanish and global linguistic competence curricula as part of health equity-conscious undergraduate medical education.

## Abbreviations Used

AAMC: Association of American Medical Colleges
LCME: Liaison Committee on Medical Education
SP: standardized patient
UICOM: University of Illinois College of Medicine
UTMB: University of Texas Medical Branch
